# Sepsis Program Activities in Acute Care Hospitals — National Healthcare Safety Network, United States, 2022

**DOI:** 10.15585/mmwr.mm7234a2

**Published:** 2023-08-25

**Authors:** Raymund B. Dantes, Hemjot Kaur, Beth A. Bouwkamp, Kathryn A. Haass, Prachi Patel, Margaret A. Dudeck, Arjun Srinivasan, Shelley S. Magill, W. Wyatt Wilson, Mary Whitaker, Nicole M. Gladden, Elizabeth S. McLaughlin, Jennifer K. Horowitz, Patricia J. Posa, Hallie C. Prescott

**Affiliations:** ^1^Department of Medicine, Emory University School of Medicine, Atlanta, Georgia; ^2^Division of Healthcare Quality Promotion, National Center for Emerging and Zoonotic Infectious Diseases, CDC; ^3^CACI International Inc, Atlanta, Georgia; ^4^Department of Internal Medicine, University of Michigan, Ann Arbor, Michigan; ^5^Office of the Chief Nurse Officer, Adult Hospitals, University of Michigan, Ann Arbor, Michigan.

**Summary**

**What is already known about this topic?**

Sepsis is a life-threatening organ dysfunction contributing to at least 350,000 deaths annually in the United States. Sepsis care is complex, requiring multidisciplinary coordination within a hospital.

**What is added by this report?**

In 2022, 73% of hospitals reported having a sepsis program, ranging from 53% among hospitals with 0–25 beds to 95% among hospitals with >500 beds. Only 55% of all hospitals provide sepsis program leaders with dedicated time to manage a sepsis program and conduct daily activities.

**What are the implications for public health practice?**

Opportunities exist to increase institutional support and improve the structure of hospital-based sepsis programs, which is the focus of CDC’s Hospital Sepsis Program Core Elements.

## Abstract

Sepsis, life-threatening organ dysfunction secondary to infection, contributes to at least 1.7 million adult hospitalizations and at least 350,000 deaths annually in the United States. Sepsis care is complex, requiring the coordination of multiple hospital departments and disciplines. Sepsis programs can coordinate these efforts to optimize patient outcomes. The 2022 National Healthcare Safety Network (NHSN) annual survey evaluated the prevalence and characteristics of sepsis programs in acute care hospitals. Among 5,221 hospitals, 3,787 (73%) reported having a committee that monitors and reviews sepsis care. Prevalence of these committees varied by hospital size, ranging from 53% among hospitals with 0–25 beds to 95% among hospitals with >500 beds. Fifty-five percent of all hospitals provided dedicated time (including assigned protected time or job description requirements) for leaders of these committees to manage a program and conduct daily activities, and 55% of committees reported involvement with antibiotic stewardship programs. These data highlight opportunities, particularly in smaller hospitals, to improve the care and outcomes of patients with sepsis in the United States by ensuring that all hospitals have sepsis programs with protected time for program leaders, engagement of medical specialists, and integration with antimicrobial stewardship programs. CDC’s Hospital Sepsis Program Core Elements provides a guide to assist hospitals in developing and implementing effective sepsis programs that complement and facilitate the implementation of existing clinical guidelines and improve patient care. Future NHSN annual surveys will monitor uptake of these sepsis core elements.

## Introduction

Sepsis, life-threatening organ dysfunction secondary to infection (*1*), contributes to at least 1.7 million adult hospitalizations and at least 350,000 deaths annually in the United States (*2*). Hospital quality improvement programs focused on sepsis have been associated with reductions in mortality, length of stay, and health care costs (*3*,*4*). In 2023, CDC has published the new Hospital Sepsis Program Core Elements (*5*) (Sepsis Core Elements), a guide to help hospitals develop multiprofessional programs to monitor and optimize early identification, management, and outcomes of sepsis.

CDC’s National Healthcare Safety Network (NHSN)* is the nation’s most widely used surveillance system for tracking patient and health care personnel safety measures, such as prevention of health care–associated infections. Hospitals reporting data to NHSN are required to complete an annual survey with questions regarding patient volume, laboratory practices, patient safety practices, and facility characteristics used in risk adjustment for quality measures.^†^ Questions regarding hospital sepsis program practices were added to the 2022 NHSN annual survey to evaluate baseline practices.

## Methods

All U.S. hospitals (approximately 6,129) are eligible to enroll in NHSN (*6*). Enrolled hospitals were required to complete the 2022 NHSN Patient Safety Component Annual Hospital Survey by March 1, 2023. Hospital staff members completed the survey electronically, on the basis of hospital practices during 2022, using the NHSN web-based application. Responses were provided to four required questions and to three additional required questions, conditional upon responses to the initial questions. The first question asked about the presence of a committee that monitors and reviews sepsis care and outcomes (sepsis committees), followed by three conditional questions regarding the functions of and staff member representation on the committee. The following three questions asked about leadership support for sepsis-related activities, approaches to rapid sepsis identification, and sepsis management protocols. Survey respondents were instructed to consult with persons leading sepsis efforts or other local expertise as needed to accurately complete the survey. Descriptive analysis, stratified by hospital size (number of beds), was completed on a data set generated on June 1, 2023, using SAS (version 9.4; SAS Institute). This activity was reviewed by CDC and was conducted consistent with applicable federal law and CDC policy.^§^

## Results

As of June 1, 2023, among 5,397 hospitals enrolled in the NHSN Patient Safety Component, 5,228 had completed the survey. Seven surveys were excluded because of incomplete responses, which resulted in inclusion of 5,221 hospitals in the analysis (97% completion rate) (Table 1). Among these hospitals, 3,787 (73%) reported having a sepsis committee. These committees were least common in hospitals with 0–25 beds (53%), and progressively more prevalent as hospital size increased (Table 2). Antimicrobial stewardship and infectious disease representatives were integrated into 55% and 45% of sepsis committees, respectively. Monitoring and review of antimicrobial use in sepsis care was reported for 61% of sepsis committees.

**TABLE 1:** Hospitals completing annual survey — Patient Safety Component, Annual Hospital Survey, National Healthcare Safety Network, United States, 2022

Hospital size, no. of beds	No. (%) of hospitals*
0–25	1,580 (30)
26–50	618 (12)
51–100	703 (13)
101–250	1,301 (25)
251–500	759 (15)
>501	260 (5)
**Total**	**5,221 (100)**

* Among 5,397 National Healthcare Safety Network–enrolled hospitals (overall 97% completion rate).

**TABLE 2:** Sepsis committee utilization, responsibilities, and representation in acute care hospitals — Patient Safety Component, Annual Hospital Survey, National Healthcare Safety Network, United States, 2022

Survey questions and responses	% of facilities responding						
	All hospitals N = 5,221	Hospital size, no. of beds					
		0–25 n = 1,580	26–50 n = 618	51–100 n = 703	101–250 n = 1,301	251–500 n = 759	>501 n = 260
Total, %	100	30	12	13	25	15	5
Our facility has a committee charged with monitoring and reviewing sepsis care and/or outcomes,* no. (%)	**3,787 (73)**	831 (53)	409 (66)	542 (77)	1,088 (84)	671 (88)	246 (95)
**Responsibilities of this committee, % of facilities^†,§,¶^**							
Monitor and review compliance with CMS SEP-1 measure	**84**	77	85	86	87	85	83
Monitor and review effectiveness of early sepsis identification strategies	**82**	77	77	82	85	86	87
Update sepsis identification and management protocols based on current evidence	**81**	77	78	80	84	85	84
Monitor and review outcomes among patients with sepsis	**81**	78	79	81	83	85	82
Develop educational materials for facility staff to improve sepsis care	**79**	72	75	79	82	84	83
Monitor and review antimicrobial use in sepsis care	**61**	59	56	58	64	65	62
**Hospital location or service representation of this committee, % of facilities^†,§,¶^**							
Emergency department	**85**	83	80	84	87	90	86
Hospital medicine	**76**	73	71	77	78	81	75
Neonatal intensive care	**6**	2	2	6	7	12	13
Critical care or intensive care	**65**	31	57	72	78	80	83
Labor and delivery	**17**	11	18	18	17	22	23
Pediatrics	**11**	7	9	10	9	16	20
Infectious disease	**45**	39	42	40	48	49	52
Antimicrobial stewardship	**55**	61	46	52	55	54	54
Infectious disease or antimicrobial stewardship**	**65**	69	60	61	65	65	64
Pharmacy	**71**	73	65	70	72	73	68
Laboratory	**55**	55	50	57	59	55	46
Information technology	**41**	28	34	40	45	48	55
Other	22	21	22	21	23	22	26

**Abbreviation:** CMS = Centers for Medicare & Medicaid; SEP-1** = **CMS Severe Sepsis and Septic Shock: Management Bundle.

* Required survey question completed by all hospitals that submitted a 2022 Annual Hospital Survey; affirmative responses are shown.

^†^ Conditional required survey question completed by facilities that answered in the affirmative to the required question.

^§^ Numerator is the number of facilities with a committee that reported a responsibility or type of representation; denominator is the number of facilities with a committee (responded in the affirmative to the required question) (example: 3,180 / 3,787 × 100 =  84%).

^¶^ Hospitals could select more than one response per question.

** Hospitals that responded with either infectious disease or antimicrobial stewardship representation, or both.

Approximately one half (55%) of all hospitals (range = 35% [0–25 beds] to 78% [>500 beds]) reported that hospital leadership provided leaders of committees supervising sepsis activities with dedicated time as required to lead these activities as part of their job description or granted or assigned protected time from their other clinical or other job responsibilities to dedicate to sepsis activities (Table 3). Other indications of leadership support for hospital sepsis programs, such as data analytic or information technology resources, were reported more commonly by larger hospitals.

**TABLE 3:** Sepsis leadership, rapid identification, and management practices in acute care hospitals — Patient Safety Component, Annual Hospital Survey, National Healthcare Safety Network, United States, 2022

Survey questions and responses	% of facilities responding						
	All hospitals N = 5,221	Hospital size, no. of beds					
		0–25 n = 1,580	26–50 n = 618	51–100 n = 703	101–250 n = 1,301	251–500 n = 759	>501 n = 260
**Total, %**	**100**	**30**	**12**	**13**	**25**	**15**	**5**
Facility leadership has demonstrated commitment to improving sepsis care*^,†^							
Providing sepsis program leaders dedicated time to manage a sepsis program and conduct daily activities	**55**	35	49	59	65	73	78
Allocating resources (e.g., information technology or data analyst support, training for stewardship team) to support sepsis efforts	**65**	47	56	69	75	83	89
Having a senior executive who serves as a point of contact or champion to help ensure the program has resources and support to accomplish its mission	**60**	40	50	62	71	79	85
Presenting information on sepsis activities and outcomes to facility leadership and/or board at least annually	**71**	52	65	77	82	88	88
Ensuring the sepsis program has an opportunity to discuss resource needs with facility leadership or board, at least annually	**60**	40	52	62	71	78	83
Communicating to staff members about sepsis activities, via email, newsletters, events, or other avenues	**70**	56	61	75	78	82	83
Providing opportunities for hospital staff training on sepsis protocols	**74**	61	66	78	81	85	87
Ensuring that staff members from key support departments and groups (e.g., information technology and emergency medicine) are contributing to sepsis activities	**70**	49	62	74	80	89	92
None of the above	**12**	20	18	10	7	3	2
Our facility uses the following approaches to assist in the rapid identification of patients with sepsis, % of facilities*^,†^							
EHR-generated alert based on SIRS criteria	**65**	58	58	65	70	76	75
EHR-generated alert based on qSOFA	**13**	10	14	12	13	17	18
EHR-generated alert based on a predictive model	**33**	21	28	30	39	45	54
EHR-generated alert using other criteria not already specified	**15**	10	11	15	18	21	27
Manual screening (e.g., use of a checklist) using SIRS or similar criteria	**47**	41	48	51	50	49	38
No standardized process	**10**	15	15	9	6	3	1
Other^§^	**5**	4	5	4	6	6	8
Our facility uses the following approaches to assist in the management of patients with sepsis, % of facilities*^,†^							
Protocols that help identify and tailor care for patients with septic shock (e.g., vasopressor orders)	**79**	65	73	82	88	90	94
Protocols that prompt the ordering of sepsis diagnostic tests such as blood cultures, lactate, urinalysis, chest radiography, etc.	**85**	76	78	88	91	94	97
Protocols that prompt the ordering of preferred antimicrobial treatment regimens for sepsis or underlying infection types	**77**	64	70	78	84	88	92
Protocols that prompt the ordering of intravenous fluids	**80**	69	75	83	86	89	92
Protocols that prompt the reassessment of resuscitative efforts	**64**	51	60	65	70	74	80
Protocols that are tailored to specific populations (e.g., neonates, pregnant, oncology, or neutropenic patients, etc.)	**34**	21	28	34	40	47	57
Automated systems (e.g., EHR timers, prompts, or dashboards) that facilitate compliance with time sensitive aspects of sepsis care	**46**	32	39	45	53	62	70
No standardized sepsis protocols or automated systems for sepsis care prompting or monitoring	**10**	17	15	9	6	3	1
Other systematic approach^§^	**4**	4	4	4	4	4	5

**Abbreviations:** EHR = electronic health record; qSOFA = quick sequential organ failure assessment; SIRS = systemic inflammatory response syndrome.

* Required survey question completed by all hospitals that submitted a 2022 Annual Facility Survey.

^†^ Hospitals could select more than one response per question.

^§ ^This included a free-text option and because of low response rate was not included in analysis.

Hospitals reported using various approaches to rapidly identify patients with sepsis; the most frequent (65%) was electronic health record–generated alerts based on systemic inflammatory response syndrome criteria (*7*), followed by manual screening (47%), and predictive models (33%). Ten percent of hospitals reported having no standardized process for assisting with rapid sepsis identification. Having no standardized process was more common in hospitals with 0–25 beds (15%) than in hospitals with >500 beds (1%).

Hospitals frequently reported the existence of protocols to assist in the management of sepsis care, including those that prompt the ordering of diagnostic tests (85%), followed by those that prompt the ordering of intravenous fluids (80%), those that identify and tailor care for septic shock (79%), and those that prompt the ordering of preferred antimicrobials for sepsis or underlying infection (77%). Sepsis protocols tailored to specific patient populations were available in one third (34%) of hospitals, ranging from 21% among hospitals with 0–25 beds to 57% among those with >500 beds. Overall, 10% of hospitals reported having no standardized protocol to assist in the management of sepsis care. Having no standardized protocol to assist in the management of sepsis care was more common in hospitals with 0–25 beds (17%) than those with >500 beds (1%).

## Discussion

This survey of the majority of U.S. hospitals describes the current state of sepsis programs and identifies potential areas of improvement. Although sepsis committees are present in most hospitals, they occur less frequently in smaller hospitals, which might have access to fewer personnel and specialty resources. Further, just over one half of responding hospitals reported that dedicated time or assigned protected time was provided to sepsis program leadership. This survey highlights opportunities to further improve the institutional support and structure of hospital-based sepsis care.

Sepsis care is complex and requires coordination across multiple clinical disciplines and hospital care locations (e.g., emergency departments, intensive care units, and hospital wards). Evidence-based care guidelines (*8*), along with state-based (e.g., New York State Department of Health Sepsis Regulations)^¶^ and federal initiatives (e.g., Centers for Medicare & Medicaid Services Severe Sepsis and Septic Shock: Management Bundle) (*9*) have emphasized the importance of protocols for early sepsis identification and prompt management. This survey demonstrated that most U.S. hospitals report having some tools and protocols for sepsis detection and early management. To achieve further improvements in sepsis care for patients throughout hospitalization and after discharge, CDC has developed Sepsis Core Elements (*5*). Sepsis Core Elements will provide a guide for creating, structuring, and resourcing comprehensive sepsis programs, so that hospitals can provide optimal sepsis care. Sepsis Core Elements are intended as a manager’s guide to complement and support the implementation of existing sepsis guidelines.

Sepsis Core Elements was modeled after CDC’s Core Elements of Hospital Antibiotic Stewardship Program (ASP),** (*5*) which provides a framework for structuring ASPs that lead to improvements in antibiotic prescribing and reductions in length of hospitalization (*10*). In the 2022 NHSN survey, approximately one half of sepsis programs reported involvement of ASPs. This survey also indicated that only 61% of sepsis committees monitor and review antimicrobial use in sepsis care, although these responsibilities might overlap with those of ASPs. Sepsis Core Elements recommends inclusion of ASP personnel on sepsis committees to facilitate rapid and optimized antimicrobial use in sepsis and discontinuation of antibiotics when underlying infection has been ruled out. Coordination and other respective ASP and sepsis program practices will continue to be tracked in future NHSN annual surveys.

### Limitations

The findings in this report are subject to at least five limitations. First, the survey is limited to acute care hospitals enrolled in NHSN and might not reflect practices among all U.S. acute care hospitals; however, hospitals enrolled in NHSN represent at least 88% of U.S. acute care hospitals (*5*). Second, although hospitals reported whether specialty services such as pediatrics and labor and delivery were included in sepsis committees, these services are not within the scope of practice at all hospitals, and thus conclusions cannot be made regarding the frequency with which these services might be missing or absent from sepsis committees. Third, although many sepsis committees do not monitor antimicrobial use in sepsis, these responsibilities overlap with those of ASPs. Collaboration among sepsis programs and ASPs is emphasized in Sepsis Core Elements to ensure optimal antimicrobial use in treating sepsis. Fourth, NHSN surveys were self-reported, and answers were not independently confirmed. Finally, this survey did not strictly define criteria for a sepsis program and is subject to respondent interpretation. Sepsis Core Elements defines specific components of sepsis programs that will be tracked in future surveys.

### Implications for Public Health Practice

These data highlight opportunities, particularly in smaller hospitals, to improve the early identification of, care for, and outcomes among patients with sepsis in the United States by ensuring that all hospitals have sepsis programs with protected time for program leaders, engagement of medical specialists, and integration with ASPs. Sepsis Core Elements provides a guide to assist hospitals in developing and implementing effective sepsis programs. Future NHSN annual surveys will monitor implementation of these sepsis core elements.

## Footnotes

*
https://www.cdc.gov/nhsn/about-nhsn/index.html

†
https://www.cdc.gov/nhsn/forms/57.103_pshospsurv_blank.pdf

§ 45 C.F.R. part 46, 21C.F.R. part 56; 42 U.S.C. Sect. 241(d); 5 U.S.C. Sect. 552a; 44 U.S.C. Sect. 3501 et seq.

¶
https://www.health.ny.gov/regulations/public_health_law/section/405/

**
https://www.cdc.gov/antibiotic-use/core-elements/index.html https://www.health.ny.gov/regulations/public_health_law/section/405/
